# Post-Thyroidectomy Development of Posterior Reversible Encephalopathy Syndrome (PRES) Due to Calcium Over-Replacement

**DOI:** 10.1210/jcemcr/luad116

**Published:** 2023-09-25

**Authors:** Olga Papalou, Ekaterini Tavernaraki, Stylianos Tsagarakis, Dimitra Argyro Vassiliadi

**Affiliations:** Department of Endocrinology Diabetes and Metabolism, National Expertise Center for Rare Endocrine Disorders, Evangelismos Hospital, Athens 10676, Greece; Department of Radiology, Evangelismos Hospital, Athens 10676, Greece; Department of Endocrinology Diabetes and Metabolism, National Expertise Center for Rare Endocrine Disorders, Evangelismos Hospital, Athens 10676, Greece; Department of Endocrinology Diabetes and Metabolism, National Expertise Center for Rare Endocrine Disorders, Evangelismos Hospital, Athens 10676, Greece

**Keywords:** posterior reversible encephalopathy syndrome, hypercalcemia, milk-alkali syndrome, postoperative hypoparathyroidism

## Abstract

Posterior reversible encephalopathy syndrome (PRES) represents a distinct neurological entity characterized by a range of neurological signs and symptoms (seizures, headache, visual abnormalities, altered consciousness, and/or focal neurological signs) and typical neuroimaging findings reflecting reversible subcortical vasogenic edema, usually in the setting of blood pressure fluctuations, cytotoxic drugs, autoimmune disorders, and eclampsia. Here we present a case of a 61-year-old woman, with a history of recent total thyroidectomy and postoperative hypoparathyroidism, who was admitted to the Emergency Department with generalized seizures. Although in this clinical setting, hypocalcemia is expected as the most possible underlying pathogenic factor for triggering seizures, the patient was diagnosed with iatrogenic hypercalcemia and milk-alkali syndrome. A brain magnetic resonance imaging (MRI) demonstrated cortical swelling and fluid-attenuated inversion recovery (FLAIR) signal abnormalities in both occipital, parietal, and right frontal lobes, consistent with PRES. The patient’s encephalopathy resolved after resolution of hypercalcemia; she had no neurological deficits on discharge, while she was restarted on lower doses of calcium for hypoparathyroidism. This case illustrates the challenges imposed by postoperative hypoparathyroidism and highlights that PRES is a rare but serious complication of hypercalcemia of which endocrinologists should be aware.

## Introduction

Posterior reversible encephalopathy syndrome (PRES) is a neurological entity, first described in 1996 by Hinchey and colleagues, characterized by a range of neurological signs and symptoms, such as headache, seizures, visual disturbances, altered mental status, and other focal neurological deficits and by neuroimaging findings of reversible subcortical vasogenic edema, typically involving the bilateral parieto-occipital regions [1]. Since its first description and in parallel with the increasing use of brain magnetic resonance imaging (MRI), numerous case reports and case series of PRES have been published, but its incidence remains still elusive. PRES commonly occurs in the setting of blood pressure fluctuations, cytotoxic drugs, autoimmune disorders, and pre-eclampsia/eclampsia, while its pathogenesis remains inadequately understood with endothelial dysfunction or damage being acknowledged as a pivotal pathophysiological mechanism [2]. In the majority of patients, both clinical and imaging manifestations are usually reversible within a period of days to months, after removal of the inciting factor. However, a considerable percentage of patients with PRES require intensive care monitoring and treatment due to severe complications such as status epilepticus, cerebral ischemia, intracerebral hemorrhage, or intracranial hypertension [3].

Hypercalcemia has also been identified as a rare cause of PRES. Notably, fewer than 20 cases are reported in the literature, with the majority of them being associated with primary hyperparathyroidism and malignancy. Herein, we describe a female patient with clinical and imaging findings indicative of PRES, due to iatrogenic hypercalcemia, as a result of post-thyroidectomy excessive oral calcium supplementation, in the context of postoperative hypoparathyroidism and milk-alkali syndrome.

## Case Presentation

A 61-year-old female was admitted to the Emergency Department with generalized seizures. She reported general malaise, anorexia, headache, and multiple episodes of vomiting (>5) that started about 12 hours before admission. Her family found her unconscious (last communication with her was estimated to be 4-5 hours ago), with gaze fixation and not verbally reacting, and she had the first episode of generalized seizures during her transfer to the hospital. She had no prior history of seizures, head trauma, hypertension, cancer, atherosclerotic disease, kidney disease, or neurological disease. She underwent a total thyroidectomy 2 weeks prior, as advised by her attending endocrinologist. This recommendation was based on the presence of a single, 2.7-cm inhomogeneous solid thyroid nodule located in the isthmus that was submerging under the manubrium. She had negative thyroid autoantibodies and no history of other autoimmune diseases. Symptoms of hypocalcemia became evident on the third postoperative day and its development was verified biochemically. She was diagnosed with postoperative hypoparathyroidism and was prescribed oral calcium carbonate (3 grams per day) along with alpha calcidol (3 micrograms per day). Notably, her calcium levels measured 5 days prior to her admission were within the upper range of normal.

Upon her admission, she had another episode of generalized seizures that was successfully treated with intravenous diazepam. Non-contrast head computed tomography (CT) scan was negative for major findings, while on neurological examination, during the postictal phase, the patient had open eyes with left gaze fixation, she was unresponsive in verbal stimuli, and unable to conduct orders (Glascow coma score [GCS]: 6/15). Pupillary reflexes were intact, there were no focal neurologic deficits or papilledema. Laboratory workup was ordered, while the straight available arterial blood gases were indicative of primary anion gap metabolic acidosis, appropriately compensated by respiratory alkalosis and additional metabolic alkalosis (pH = 7.35, pCO2 = 33 mmHg, pO2 = 79 mmHg, HCO3^−^ = 19 mmol/L, lactate = 12 mmol/L, glucose = 188 mg/dL (10.43 mmol/L), ionized Ca = 1.35 mmol/L, Na = 146 mmol/L, K = 5.6 mmol/L). Her electrocardiogram (ECG) revealed sinus tachycardia (134 bpm), with ST depression and normal corrected QT interval = 444 msec, while her blood pressure was 140/80 mmHg.

## Diagnostic Assessment

**Figure 1.**
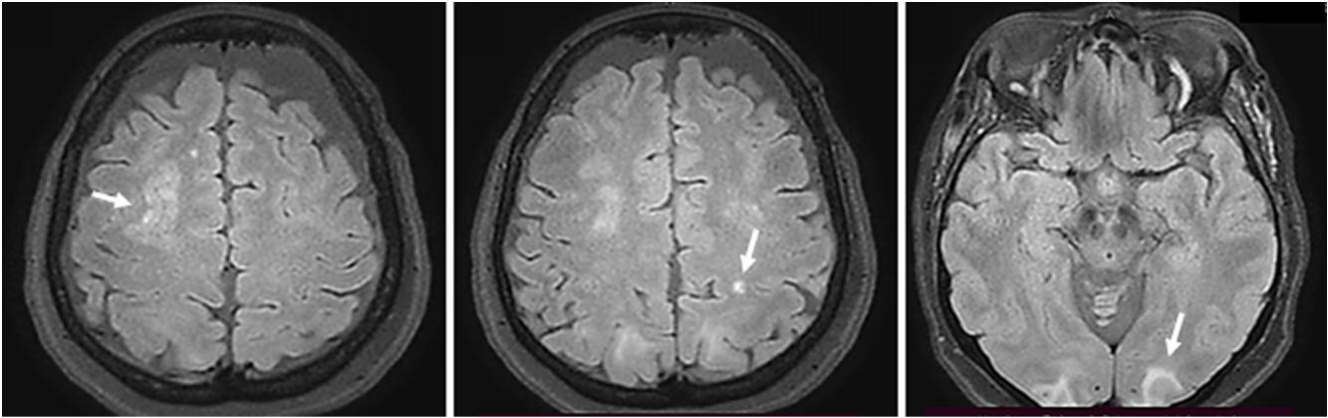
Brain MRI findings of the patient at the time of diagnosis, with bilateral cortical and subcortical hyperintense lesions (arrows) of both occipital, parietal, and right frontal lobes in T2-weighted or FLAIR sequences, diagnostic for PRES.

After her admission to the Endocrinology Department, the patient presented gradual improvement of her mental and neurological status. She started responding to verbal stimuli, initially disoriented but gradually restoring orientation in time and space; her left gaze fixation and ocular motility was resolved, but she continued reporting visual disturbances, such as diplopia and visual hallucinations. Twenty-four hours after her admission, other than residual dysmetria on “finger-to-nose” exam and difficulty with fine motor tasks, the patient had an otherwise normal neurological examination. A full neurologic workup was conducted. Lumbar puncture was normal, while her electroencephalogram (EEG) was disorganized, with intermittent α rhythm recording in the posterior occipital regions bilaterally and frequent intermittent spiking bradyrhythmias θ and δ of mean potential as a possible indication of multiple subcortical lesions and/or encephalopathic disorder. Workup was completed with brain MRI, in which cortical swelling and fluid-attenuated inversion recovery (FLAIR) signal abnormalities of both occipital, parietal, and right frontal lobes was observed, consistent with PRES (Fig. 1).

PTH was undetectable both on admission, as well as few days later, after normalization of calcium levels, confirming hypoparathyroidism. Her serum thyroid-stimulating hormone (TSH) level was mildly elevated (TSH = 9.12 μU/mL [9.12 mU/L]) with a low free thyroxine level (fT4 = 0.4 ng/dL [5.14 pmol/L]), due to recent start of levothyroxine therapy. Phosphate, cortisol, alkaline phosphatase, and serum 25-hydroxyvitamin D were within normal limits. To exclude other causes of PRES, lab testing for autoimmune diseases, thrombophilia, hepatitis, and human immunodeficiency virus (HIV) was performed and results were unremarkable for major findings.

## Treatment

The patient’s condition was deemed critical, with near status epilepticus indicated by 2 witnessed seizure episodes, and the possibility of additional unobserved seizures given her unresponsiveness upon discovery by her family. When she was initially admitted to the Emergency Department, the available information centered around her recent thyroidectomy and postoperative hypoparathyroidism. In this context, hypocalcemia was considered as the most probable underlying triggering mechanism of seizures and administration of intravenous calcium was prioritized promptly, without waiting for the biochemical test results. However, upon receiving the initial laboratory results performed before any intervention, a surprising finding of hypercalcemia was uncovered, with a corrected calcium level of 14.4 mg/dL (3.59 mmol/L), high normal phosphate levels and suppressed parathyroid hormone (PTH) levels, and acute renal impairment (creatinine = 2.9 mg/dL [256.36 μmol/L] and estimated glomerular filtration rate [eGFR] = 18 mL/min/1.73 m^2^) (Table 1). Intravenous calcium administration was interrupted, and intravenous saline infusion was initiated, while the triad of hypercalcemia/metabolic alkalosis/acute renal failure, in the setting of calcium carbonate treatment, raised the suspicion of milk-alkali syndrome.

**Table 1.** Patient's laboratory workup upon admission

Lab parameter	Patient's value	Reference range	Lab parameter	Patient's value	Reference range
Glucose	127 mg/dL (7 mmol/L)	70-110 mg/dL (3.8-6.1 mmol/L)	γ-GT	11 U/L	7-32 U/L
Urea	102 mg/dL (36.4 mmol/L)	10-50 mg/dL (3.5-17 mmol/L)	Total Ca	15.2 mg/dL (3.79 mmol/L)	8.5-10.5 mg/dL (2.12-2.62 mmol/L)
Creatinine	2.9 mg/dL (256.36 μmol/L)	0.5-0.9 mg/dL (44.2-79.56 μmol/L)	Phosphorus	4.7 mg/dL (1.5 mmol/L)	2.5-5 mg/dL (0.8-1.6 mmol/L)
Sodium	140 mmol/L	135-147 mmol/L	Magnesium	2.27 mg/dL (0.93 mmol/L)	1.58-2.55 mg/dL (0.65-1.05 mmol/L)
Potassium	4.1 mmol/L	3.5-5.1 mmol/L	25-OH-vitamin D	21.6 ng/mL (53.9 nmol/L)	30-100 ng/mL (75-250 nmol/L)
Total protein	7.2 g/dL (72 g/L)	6-8.2 (60-82 g/L)	PTH	<3 pg/mL (<3 ng/L)	10-67 pg/mL (10-67 ng/L)
Albumin	5.0 g/dL (50 g/L)	3.5-5 g/dL (35-50 g/L)	FT4	0.4 ng/dL (5.14 pmol/L)	0.93-1.7 ng/dL (11.97-21.8 pmol/L)
SGOT	49 U/L	5-37 U/L	TSH	9.12 μU/mL (9.12 mU/L)	0.27-4.2 μU/mL (0.27-4.2 mU/L)
SGPT	21 U/L	5-40 U/L	CRP	0.8 mg/dL (8 mg/L)	<0.5 mg/dL (<5 mg/L)
ΑLP	78 U/L	35-104 U/L			

Abbreviations: ALP, alkaline phosphatase; CRP, C-reactive protein; FT4, free thyroxine; γ-GT, gamma-glutamyl transferase; PTH, parathyroid hormone; SGOT, aspartate aminotransferase (AST); SGPT, alanine aminotransferase (ALT); TSH, thyrotropin (thyroid-stimulating hormone).

## Outcome and Follow-Up

**Figure 2.**
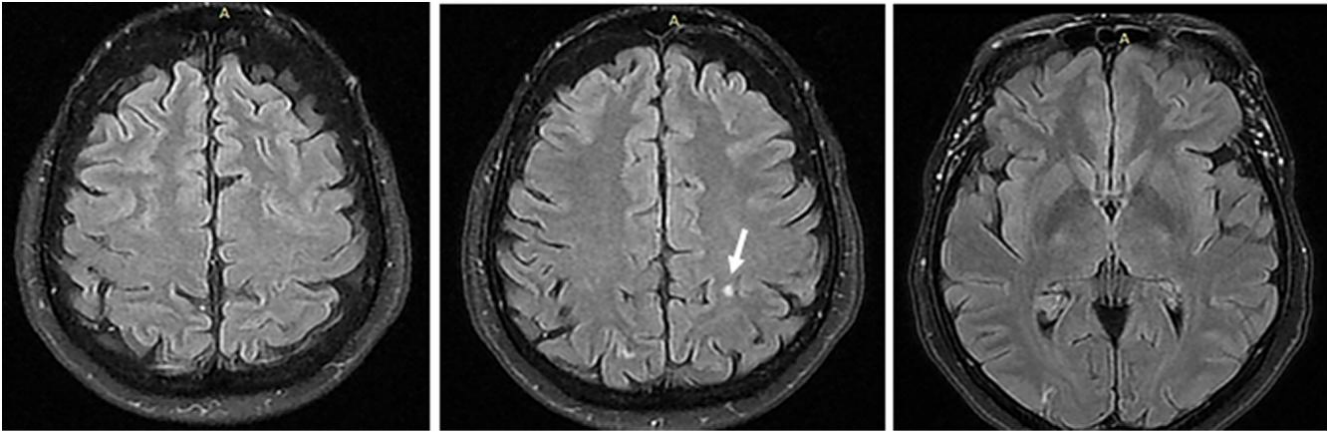
Brain MRI 3 months after the episode, with complete resolution of the previous reported abnormalities, except for a small gliotic lesion (4 mm) in the white matter of the left posterior superior parietal lobe (arrow).

The patient’s encephalopathy symptoms resolved after resolution of hypercalcemia, and she had no neurological deficits on discharge. Kidney function was restored to baseline, postsurgical creatinine levels, which were slightly elevated (creatinine = 1.3 mg/dL [114.92 μmol/L] and calculated eGFR = 44 mL/min/1.73 m^2^). Furthermore, she developed hypocalcemia and therefore lower doses of calcium were restarted. She is now on close follow-up. Use of calcium citrate was preferred, to reduce the risk for side-effects and redevelopment of milk-alkali syndrome. Repeat brain MRI, at 3 months after the episode, revealed only a small gliotic lesion (4 mm) in the white matter of the left posterior superior parietal lobe (Fig. 2).

## Discussion

PRES represents a distinct neurological entity characterized by typical neuroimaging findings and most commonly is followed by a good prognosis, when the inciting factor is removed. The most commonly encountered risk factors for PRES are hypertensive crisis, pre-eclampsia, autoimmune diseases, and cytotoxic immunosuppressive therapy. However, since its first description, various medical conditions have been also associated with PRES, including hypercalcemia. Based on the existing literature, fewer than 20 cases of hypercalcemia-associated PRES have been reported, most of them in the context of primary hyperparathyroidism and malignancy. In our case, hypercalcemia was iatrogenic, as a result of excessive oral calcium supplementation and milk-alkali syndrome in the context of postoperative hypoparathyroidism, a rather benign and commonly encountered endocrinopathy in daily clinical practice. There are only 3 similar cases found in the literature, with 2 of them manifested in a post-thyroidectomy clinical setting [4, 5] and with only one of them associated with milk-alkali syndrome [6].

PRES has been reported in almost all age groups, from children to older adults, but most frequently in young- or middle-aged adults with a preponderance in females. Pathophysiologically, there is no established underlying mechanism, but there are 3 chief theories, which mainly involve endothelial dysfunction/damage, as a result of failure of cerebral blood flow autoregulation particularly in the posterior brain regions due to toxins and/or vasospasm. Cytokine activation might also underlie the pathophysiological changes observed in PRES, which is further supported by the fact that nearly half of patients with PRES have a history of an autoimmune disorder [7]. Unsurprisingly, since calcium signaling is considered a key regulatory mechanism in endothelial cells for barrier function and inflammation, deviations in intracellular calcium concentrations can be another triggering mechanism of PRES “endotheliopathy” [8]. Renal dysfunction is frequently observed in PRES cases. Nevertheless, it remains unclear whether this impairment represents an autonomous risk factor for PRES or rather a concomitant expression of the underlying trigger, such as hypertension or autoimmune disorders.

Clinically, neurological symptoms of PRES manifest acutely or subacutely, usually developing during several hours or days. Encephalopathy ranges from mild confusion to severe altered consciousness, such as deep stupor. Generalized tonic-clonic seizures occur in the majority of patients (60%-75%), while visual abnormalities, including decreased visual acuity, visual field deficits, cortical blindness, or hallucinations, is another clinical symptom/sign that characteristically manifests in PRES and should raise our suspicion. MRI is the diagnostic gold standard in PRES, also useful in the differential diagnosis. Typically, MRI findings involve hyperintense signals in FLAIR sequences in the parieto-occipital and posterior frontal cortical and subcortical white matter, principally due to vasogenic edema. Less commonly, the brainstem, basal ganglia, and cerebellum are involved. Overall, currently there are no diagnostic criteria for PRES, and both clinical and neuroimaging findings are often not specific. Thus, exclusion of other causes and differential diagnosis is mandatory in all patients.

Postoperative hypoparathyroidism, caused by intraoperative injury to the parathyroid glands through compromising vascular supply, thermic injury, or inadvertent excision, is one of the most common complications of neck surgery, occurring transiently in 25% to 80% of patients and permanently (lasting more than 6 months) in up to 5% of cases. There is a higher risk in women, patients over the age of 60 years, and those undergoing operation in centers with lower experience. Although its management with oral calcium and vitamin D supplementation might seem easy, in real clinical practice management of these patients is actually challenging and unfortunately commonly accompanied by impaired quality of life and significant long-term morbidity. Taking into account the constantly increasing thyroid surgeries, endocrinologists should be well familiarized with the difficulties in managing postoperative hypoparathyroidism, as well as the complications that may occur, which do not only involve undertreatment and hypocalcemia but also overtreatment as in the presented case [9].

Milk-alkali syndrome or calcium-alkali syndrome, as a result of ingestion of high amounts of calcium carbonate, is characterized by the triad of hypercalcemia, metabolic alkalosis, and renal impairment. Although in the past, it was commonly associated with antacids overconsumption, contemporary use of calcium-containing supplements for treatment/prevention of osteoporosis, as well as postsurgical hypoparathyroidism, has led to a resurgence of the syndrome, estimated to be the third most common cause of hypercalcemia. In addition to excess consumption, other pathogenetic processes such as variances in calcium homeostasis and renal tubular reabsorption, abnormalities of vitamin D metabolism, and differences in alkaline secretion also play a role in the development milk-alkali syndrome, while diagnosis relies on excluding other causes of hypercalcemia [10]. In our patient, the preexisting, unknown renal impairment, for which the underlying cause remains elusive, could have acted as a catalytic factor contributing to the emergence of both milk-alkali syndrome and PRES.

Overall, this case schematically illustrates the difficulties in managing postoperative hypoparathyroidism and highlights PRES as a rare but serious complication of hypercalcemia, as well as milk-alkali syndrome as an increasingly encountered complication of calcium over-supplementation that endocrinologists should bear in mind in daily clinical practice.

## Learning Points

- PRES is a rare but increasingly encountered complication of hypercalcemia, of any origin, typically manifesting with seizures, headache, visual abnormalities, altered mental status, and/or other focal neurological deficits. Brain MRI is the diagnostic gold standard.
- Postoperative hypoparathyroidism poses great challenges in its management. Clinical endocrinologists are usually more sensitive to clinical symptoms/signs of undertreatment and hypocalcemia; however, also overtreatment may result in serious complications, such as milk-alkali syndrome and PRES. In particular, patients with chronic kidney disease may be more prone to developing the above-mentioned consequences of over-supplementation and should be more closely monitored.
- Milk-alkali syndrome, once thought to be a rare condition, is becoming more commonly diagnosed due to the contemporary use of calcium-containing supplements; it is characterized by the classic triad of hypercalcemia, metabolic alkalosis, and renal impairment.

## Contributors

O.P., D.A.V., and S.T. were involved in the diagnosis and management of this patient and manuscript submission. E.T. was involved in brain MRI imaging interpretation and preparation of MRI images.

## Data Availability

Original data generated and analyzed during this study are included in this published article.

## Abbreviations

FLAIR: fluid-attenuated inversion recovery
fT4: free thyroxine
MRI: magnetic resonance imaging
PRES: posterior reversible encephalopathy syndrome
PTH: parathyroid hormone
TSH: thyrotropin (thyroid-stimulating hormone)
